# Supplementary material for: Cytotoxic Furan- and Pyrrole-Containing Scalarane Sesterterpenoids Isolated from the Sponge Scalarispongia sp
Source: Molecules. 2019 Feb 27;24(5):840. doi: 10.3390/molecules24050840 (PMC6429051; doi:10.3390/molecules24050840)
Supplement: Supplementary file 1 [file molecules-24-00840-s001.pdf]

Supplementary Materials

Cytotoxic Furan- and Pyrrole-Containing Scalarane Sesterterpenoids Isolated from the Sponge *Scalarispongia* sp.

Yeon-Ju Lee [1,2,*], Su Hyun Kim [1,2], Hansol Choi [1], Hyi-Seung Lee [1,2], Jong Seok Lee [1,2], Hee Jae Shin [1,2], and Jihoon Lee [1,2]

[1] Marine Natural Products Chemistry Laboratory, Korea Institute of Ocean Science and Technology, 385 Haeyangro, Busan 49111, Republic of Korea

[2] Department of Marine Biotechnology, University of Science and Technology, 217 Gajungro, Daejeon 34113, Republic of Korea

* Correspondence: yjlee@kiost.ac.kr; Tel.: +82-51-664-3350

Table S1. Complete NMR data of **3** in $CDCl_3$

Figure S1. ^1H and ^{13}C NMR Spectra (500 MHz and 125 MHz, $CDCl_3$) of **3**

Figure S2. HMBC and HSQC NMR spectra of **3**

Figure S3. COSY and NOESY NMR spectra of **3**

Table S2. Complete NMR data of **4** in $CDCl_3$

Figure S4. ^1H and ^{13}C NMR Spectra (500 MHz and 125 MHz, $CDCl_3$) of **4**

Figure S5. HMBC and HSQC NMR spectra of **4**

Figure S6. COSY and NOESY NMR spectra of **4**

Table S3. Complete NMR data of **5** in $CDCl_3$

Figure S7. ^1H and ^{13}C NMR Spectra (500 MHz and 125 MHz, $CDCl_3$) of **5**

Figure S8. HMBC and HSQC NMR spectra of **5**

Figure S9. COSY and NOESY NMR spectra of **5**

Figure S10. ^1H and ^{13}C NMR Spectra (500 MHz and 125 MHz, $CDCl_3$) of **1**

Figure S11. ^1H and ^{13}C NMR Spectra (500 MHz and 125 MHz, $CDCl_3$) of **2**

Figure S12. ^1H and ^{13}C NMR Spectra (500 MHz and 125 MHz, $CDCl_3$) of **6**

Figure S13. ^1H and NMR Spectra (500 MHz, $CDCl_3$) of the crude extract obtained by the treatment of the mixture of 1 and 2 with silica and methanol

Table S1. Complete NMR data of **3** in $CDCl_3$

Position	δ_C, type	δ_H (*J* in Hz)	HMBC	COSY
1	39.9 CH_2	0.62 ,br dd (14.0, 14.0)		1.58
		1.58, m		0.62
2	18.7 CH_2	1.38, m		1.61,0.62
		1.61, m		1.38
3	42.1 CH_2	1.13, m		1.36
		1.36, m		1.13
4	33.5 C			
5	56.8 CH	0.83, m	37.2, 39.9	
6	18.2 CH_2	1.31, m		1.57
		1.57, m		1.06,1.31
7	41.2 CH_2	1.06, br dd (13.5,10.5)	37.7, 53.1, 56.8	1.06,1.31
		1.78, m		1.57,1.78,
8	37.7 C			
9	53.1 CH	1.31, m	21.9	1.81, 1.92
10	37.2 C			
11	21.9 CH_2	1.81, m		1.31, 1.92, 5.48
		1.92, m	41.7	1.31, 1.81, 5.48
12	73.0 CH	5.48, br s	172.7	1.81, 1.92
13	41.7 C			
14	50.9 CH	2.34, br d (13.5)	34.9, 37.7, 41.7, 194.6	2.47, 2.57
15	34.9 CH_2	2.47, dd (17.5,13.5)	41.7, 50.9, 120.2, 194.6	2.34, 2.57
		2.57, br d (17.5)	41.7, 50.9, 194.6	2.34, 2.47
16	194.6 C			
17	120.2 C			
18	172.7 C			
19	142.9 CH	7.26, br s	106.5, 120.2, 172.7	6.59
20	106.5 CH	6.59, br s	120.2, 142.9, 172.7	7.26
21	33.5 CH_3	0.85, s	33.5, 42.1, 56.8	
22	21.5 CH_3	0.81, s	33.5, 42.1, 56.8	
23	16.3 CH_3	0.85, s	53.1, 56.8	
24	17.0 CH_3	0.99, s	37.7, 41.2, 50.9, 53.1	
25	20.4 CH_3	1.32, s	41.7, 50.9, 73.0, 172.7	
12OAc	172.7 C			
	21.4 CH_3	1.88, s	170.2	

Figure S3. ^{1}H and ^{13}C NMR Spectra (500 MHz and 125 MHz, $CDCl_3$) of **3**

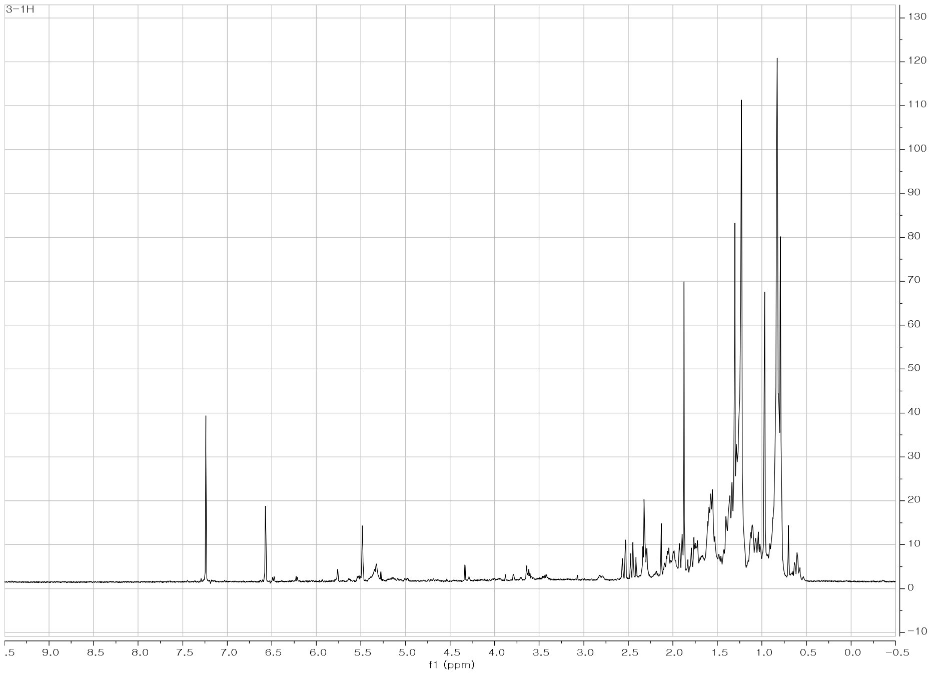

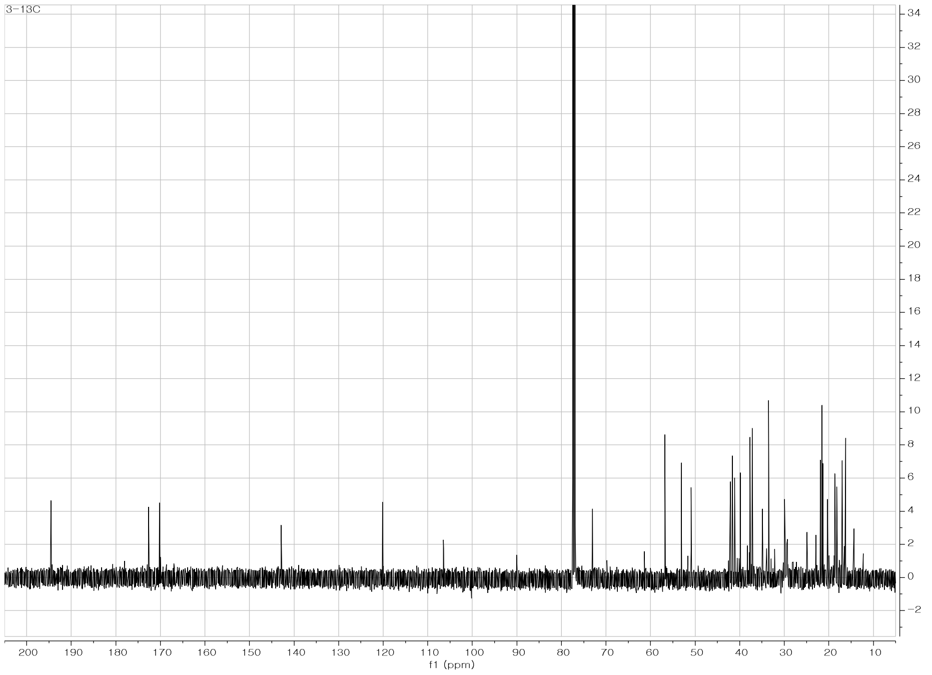

Figure S2. HMBC and HSQC NMR spectra of **3**

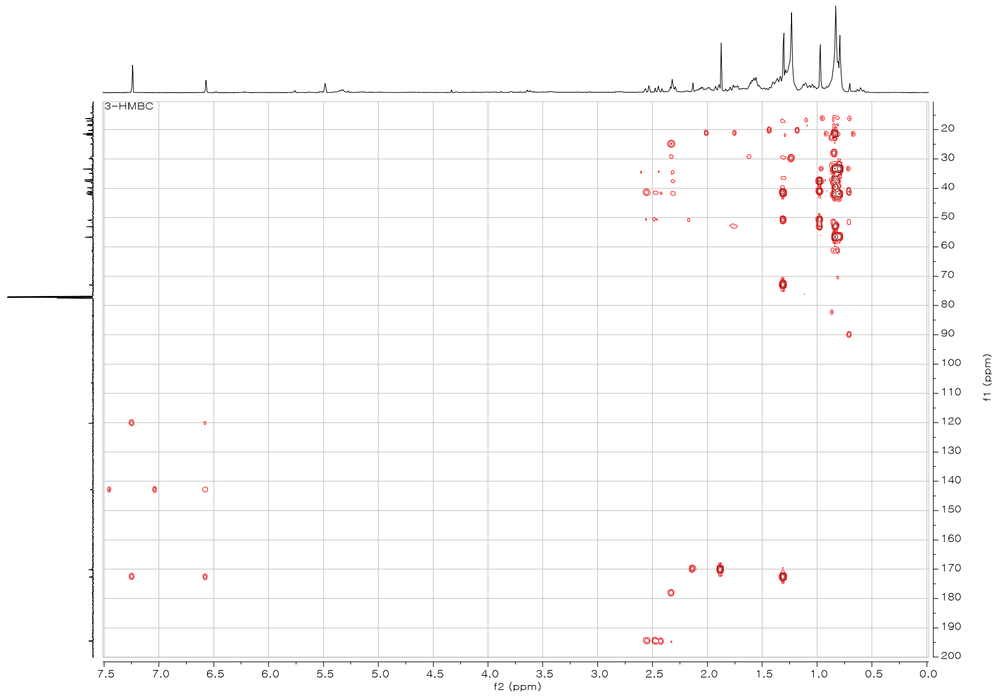

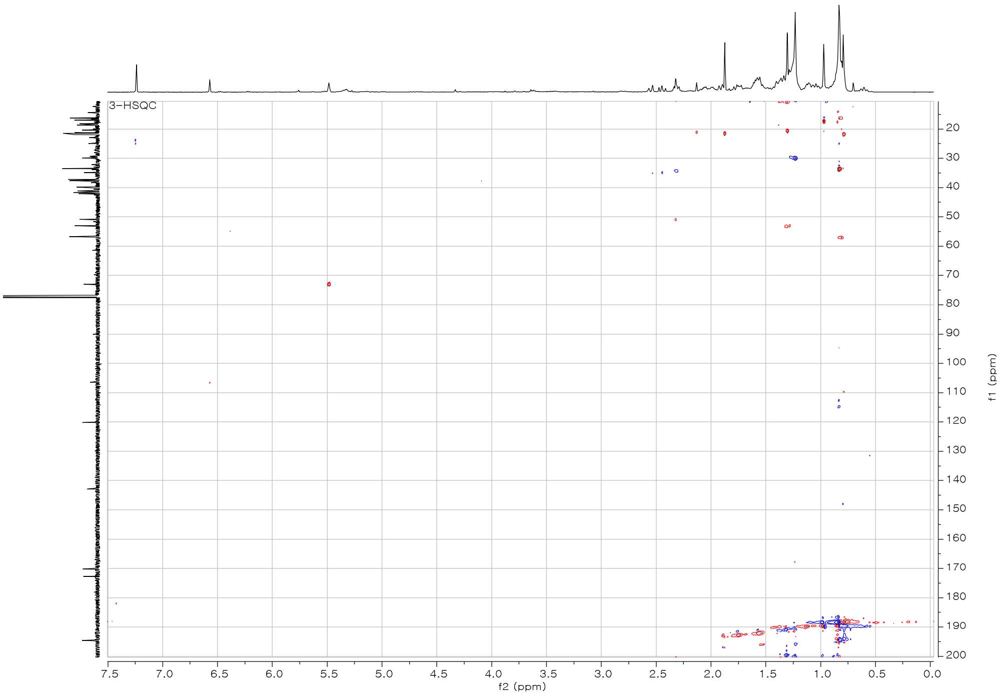

Figure S3. COSY and NOESY NMR spectra of **3**

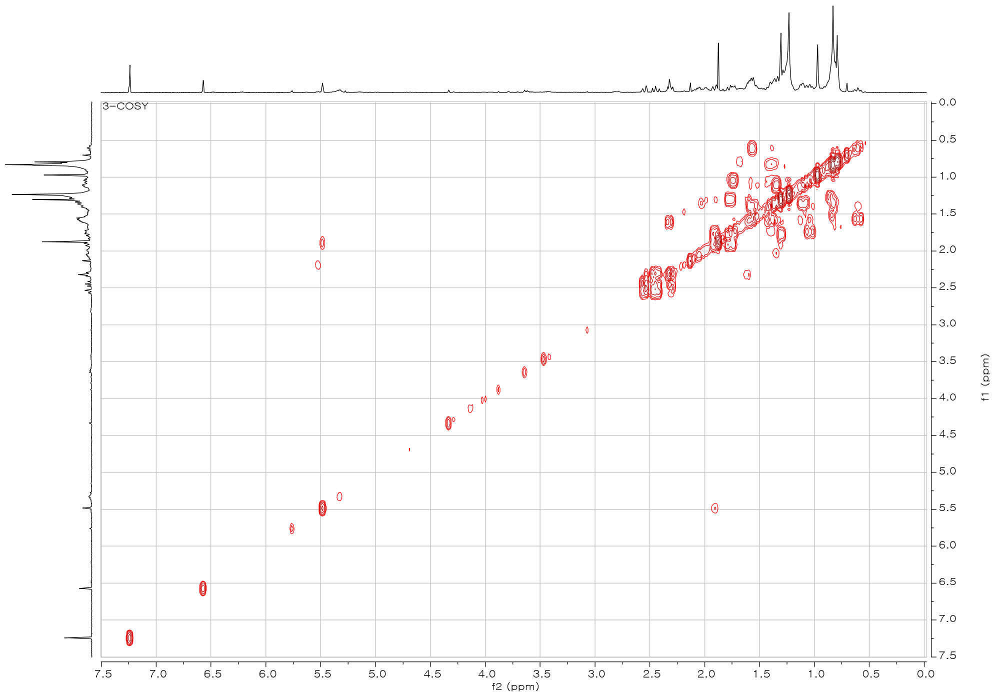

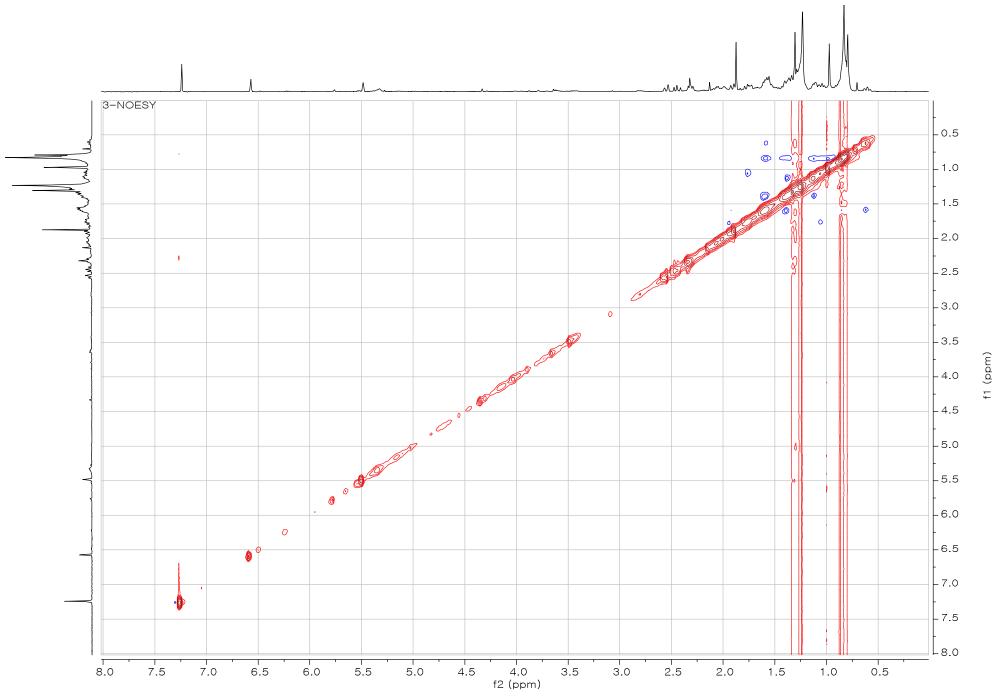

Table S2. Complete NMR data of **4** in $CDCl_3$

Position	δ_C, type		δ_H (*J* in Hz)	HMBC	COSY
1	39.9	CH_2	0.63, ddd (14.5, 14.5, 4.5)		1.39, 1.58
			1.58, m	37.2, 42.2	0.63
2	18.3	CH_2	1.39, m	37.2, 39.9	0.63, 1.56
			1.56, m		1.39
3	42.2	CH_2	1.12, ddd (13.0, 13.0, 4.0)	33.5	1.35
			1.35, m		1.12
4	33.5	C			
5	56.7	CH	0.83, m		1.40
6	18.7	CH_2	1.40, m	56.7	0.83
			1.58, m	37.2, 38.0, 56.7	1.77
7	41.4	CH_2	1.04, ddd (11.5, 11.5, 3.5)	17.1	1.77
			1.77, m	38.0, 52.7, 56.7	1.58,1.84
8	38.0	C			
9	52.7	CH	1.38, m	22.6, 51.6	1.81
10	37.2	C			
11	22.6	CH_2	1.63, m		
			1.81, m	38.0, 52.7, 74.8	1.38,5.45
12	74.8	CH	5.45, br s	52.7, 171.2	1.81
13	39.5	C			
14	51.6	CH	2.19, dd (7.3,10.0)	17.1, 25.2, 35.4, 38.0, 39.5, 41.4, 52.7, 135.5, 196.8	2.48,2.49
15	35.4	CH_2	2.48, br d (7.3)	39.5, 51.6, 121.1, 196.8	2.19
			2.49, br d (10.0)	39.5, 51.6, 121.1, 196.8	2.19
16	196.8	C			
17	121.1	C			
18	135.5	C			
19	119.5	CH	7.30, br s	111.2, 121.1, 135.5	6.32
20	111.2	CH	6.32, br s	119.5, 121.1, 135.5	7.30
21	33.5	CH_3	0.84, s	21.6, 33.5, 42.2, 56.7	
22	21.6	CH_3	0.81, s	33.5, 42.2, 56.7	
23	16.4	CH_3	0.84, s	37.2, 39.9, 52.7	
24	17.1	CH_3	0.98, s	38.0, 41.4, 51.6	
25	25.2	CH_3	1.27, s	39.5, 51.6, 74.8, 135.5	
12OAc	171.2	C			
	21.5	CH_3	1.91, s	171.2	

Figure S4. ^{1}H and ^{13}C NMR Spectra (500 MHz and 125 MHz, $CDCl_3$) of **4**

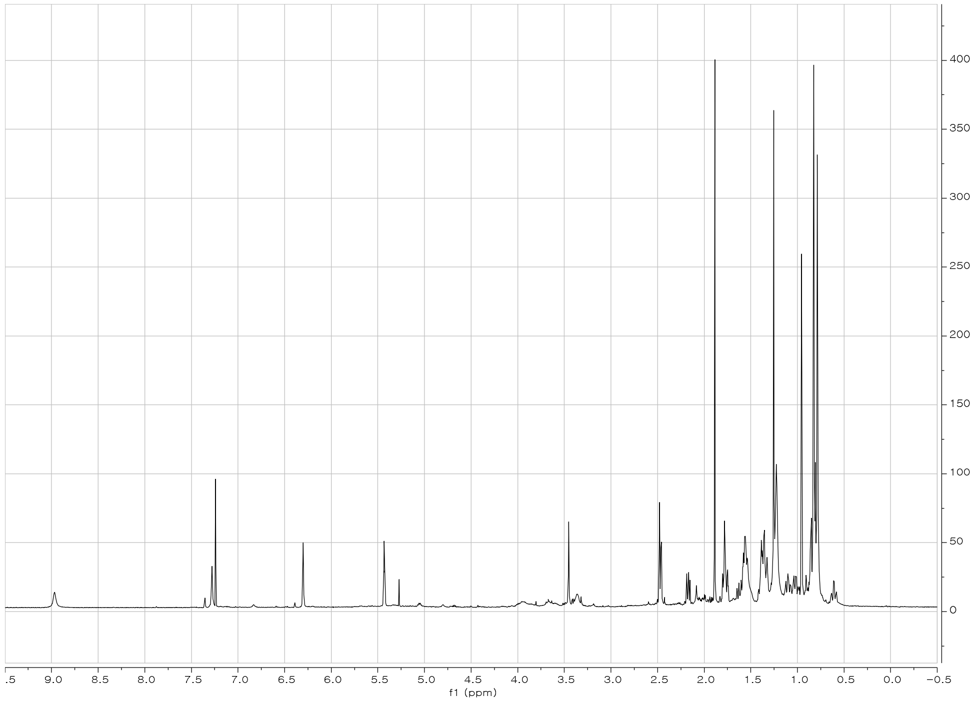

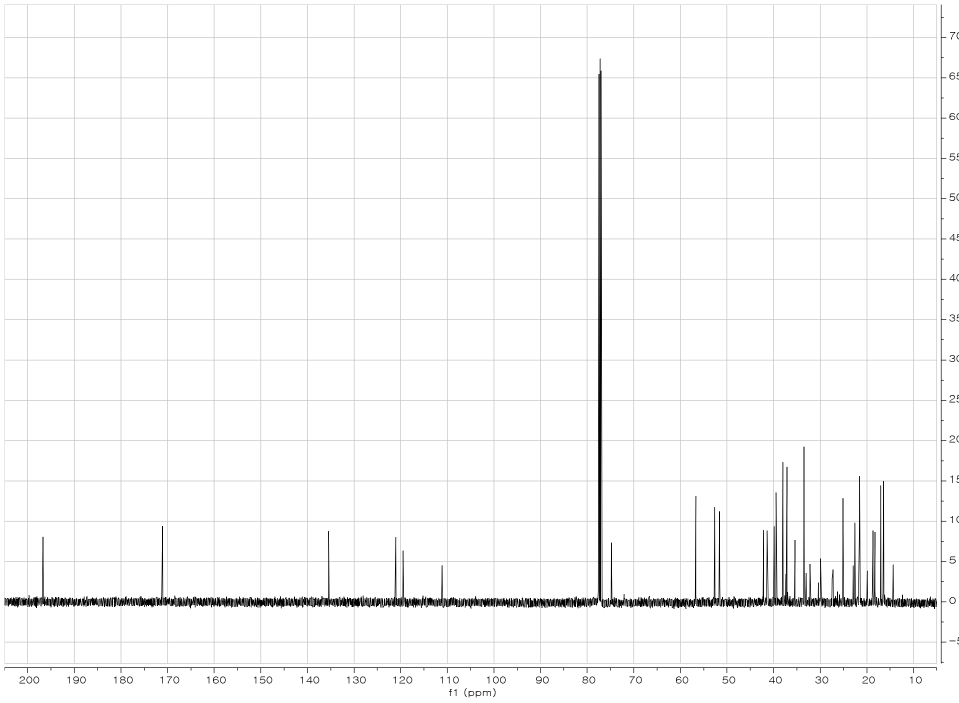

Figure S5. HMBC and HSQC NMR spectra of **4**

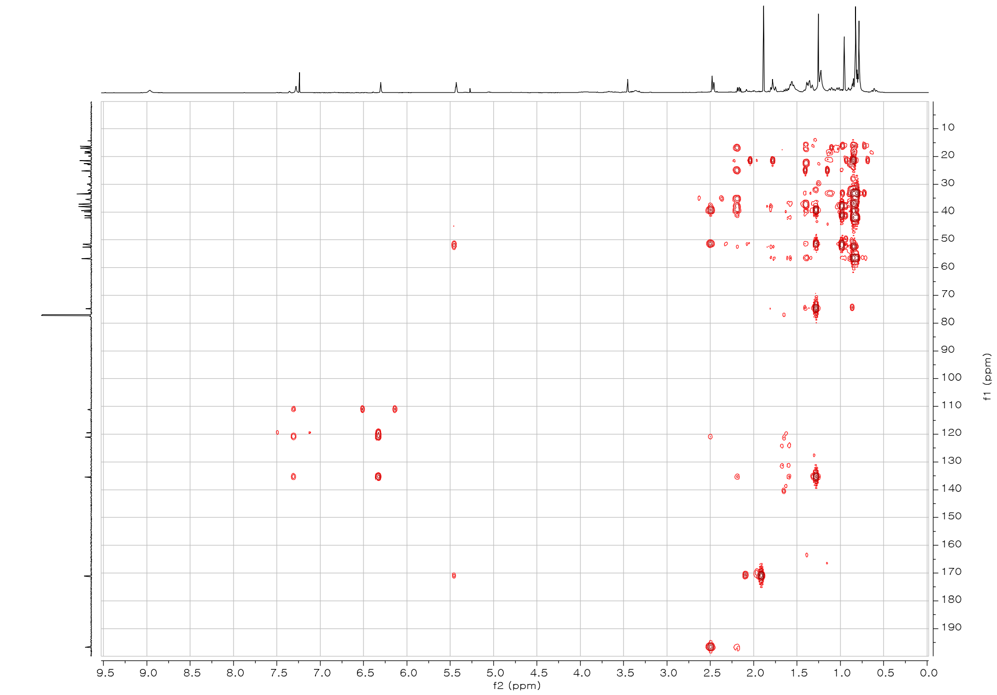

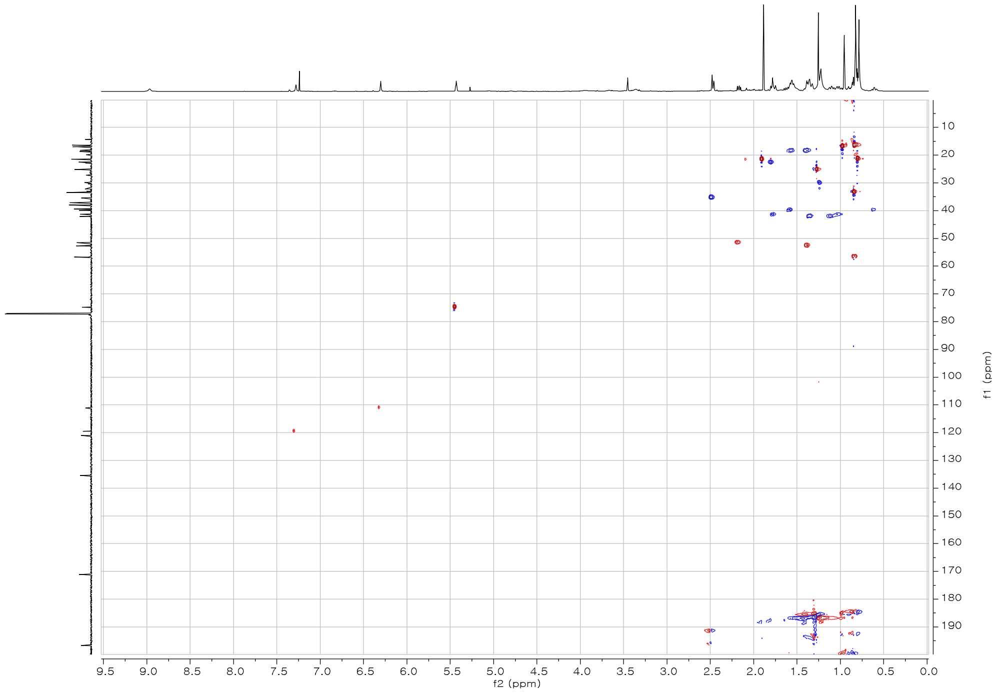

Figure S6. COSY and NOESY NMR spectra of **4**

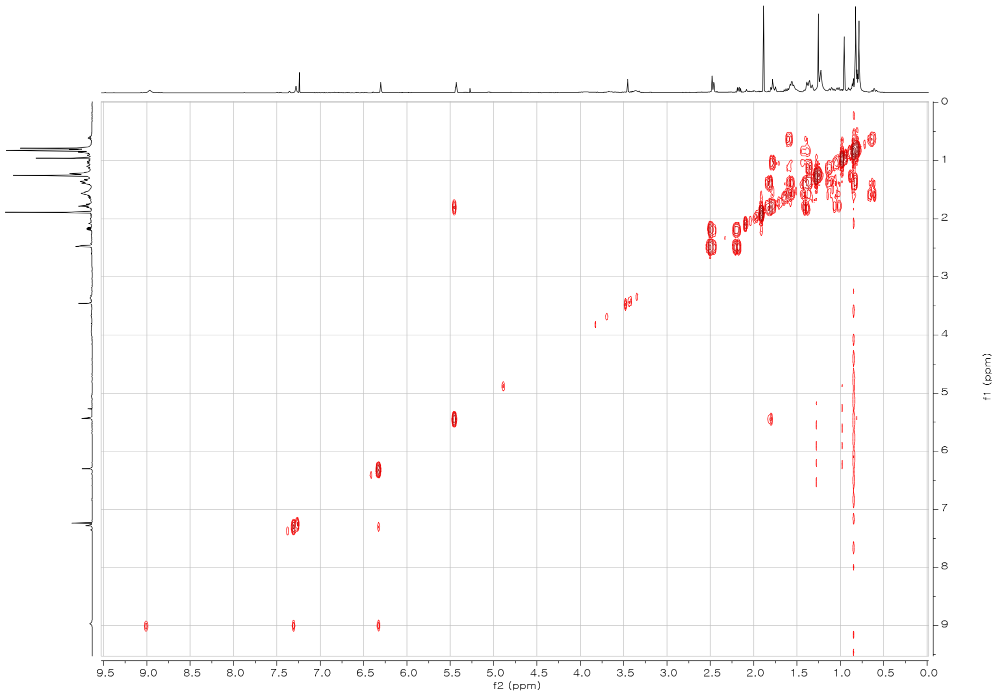

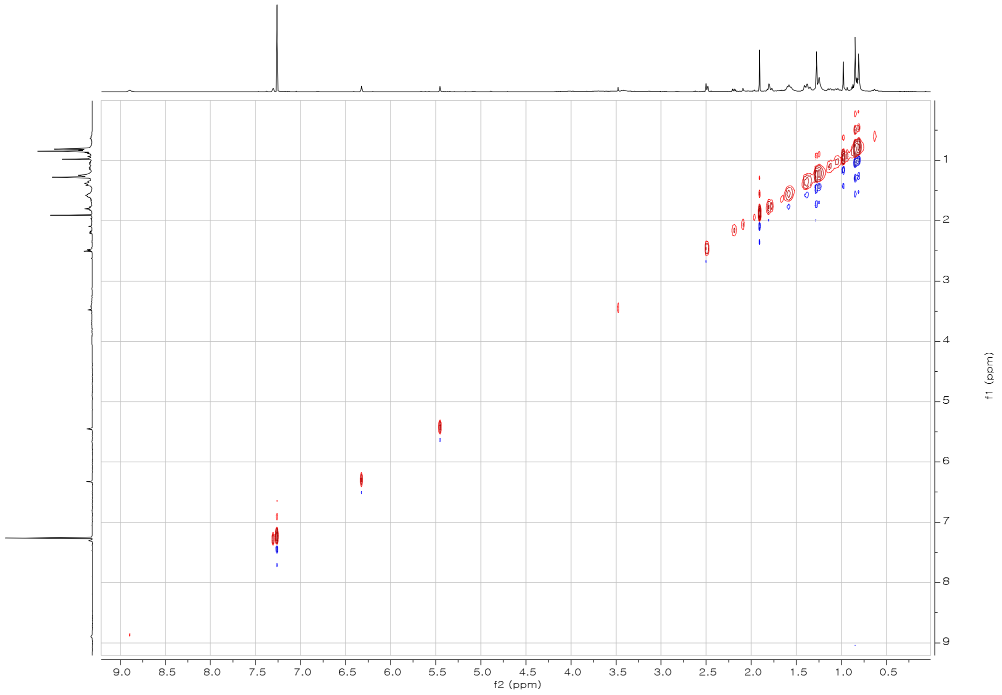

Table S3. Complete NMR data of **5** in $CDCl_3$

Position	δ_C, type	δ_H (*J* in Hz)	HMBC	COSY
1	39.9 CH_2	0.62, ddd (13.0, 13.0, 3.0)		1.40, 1.60
		1.57, m	42.2, 56.5	1.60
2	18.7 CH_2	1.40, m		0.62, 1.11, 1.60
		1.60, m		0.62, 1.11, 1.40
3	42.2 CH_2	1.11, br dd (12.5, 5.0)	21.5	1.35, 1.40, 1.60,
		1.35, m	39.9, 56.5	1.11
4	33.5 C			
5	56.5 CH	0.90, m	33.5	1.44
6	18.3 CH_2	1.44, m		0.90, 1.11, 1.60
		1.60, m	41.4	1.44, 1.82
7	41.4 CH_2	1.11, m	17.6, 37.2	1.44, 1.82
		1.82, m		1.11, 1.60
8	37.2 C			
9	53.2 CH	1.35, m	37.2, 45.9, 56.5	1.74
10	37.2 C			
11	22.0 CH_2	1.74, br d (14.0)	37.2, 53.2	1.35, 1.82, 5.42
		1.82, m	53.2	1.74, 5.42
12	73.6 CH	5.42, br s	45.9, 53.2	1.74, 1.82
13	41.2 C			
14	45.9 CH	2.14, br d (13.0)	17.6, 21.0, 23.8, 37.2	1.59
			41.2, 71.8, 158.9	
15	23.8 CH_2	1.59, m	45.9	2.07, 2.14, 4.18
		2.07, br d (15.0)	41.2, 71.8, 116.4	1.59, 4.18
16	71.8 C	4.18, d (3.5)	45.9, 116.4, 158.9	1.59, 2.07
17	116.4 C			
18	158.9 C			
19	141.0 CH	7.19, br s	110.2, 116.4, 158.9	6.28
20	110.2 CH	6.28, br s	116.4, 141.0, 158.9	7.19
21	33.5 CH_3	0.85, s	21.5, 33.5, 42.2, 56.5	
22	21.5 CH_3	0.81, s	33.5, 42.2	
23	16.2 CH_3	0.83, s	37.2, 39.9, 53.2	
24	17.6 CH_3	0.93, s	37.2, 41.4, 45.9, 53.2	
25	21.0 CH_3	1.18, s	41.2, 45.9, 73.6, 158.9	
12OAc	170.8 C			
	21.6 CH_3	1.86, s	170.8	
16OMe	56.6 CH_3	3.40, s	71.8	

Figure S7. ^{1}H and ^{13}C NMR Spectra (500 MHz and 125 MHz, $CDCl_3$) of **5**

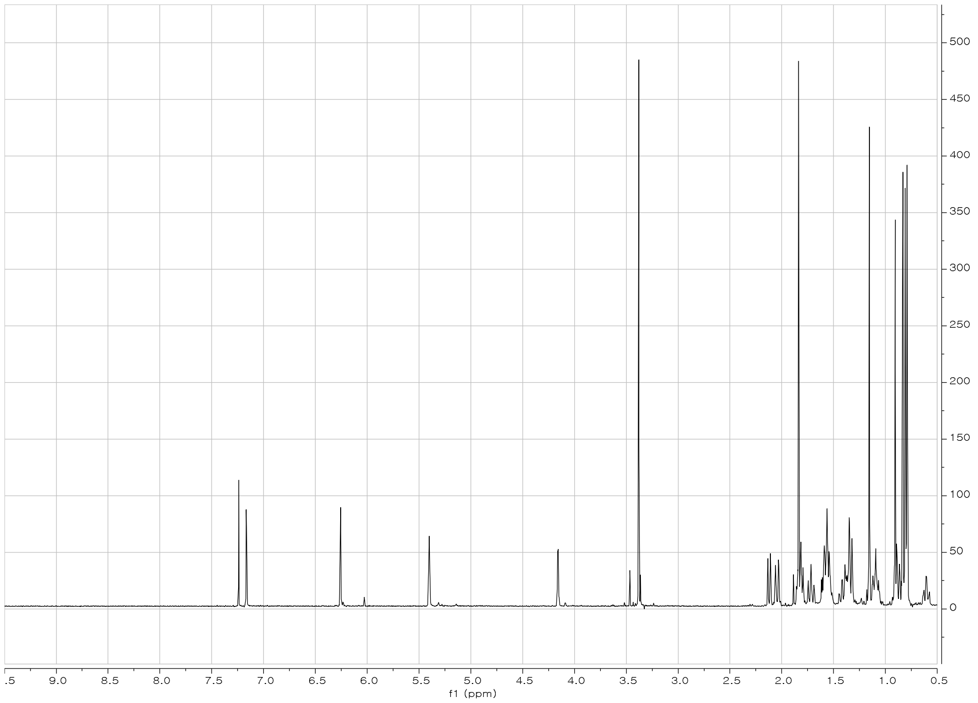

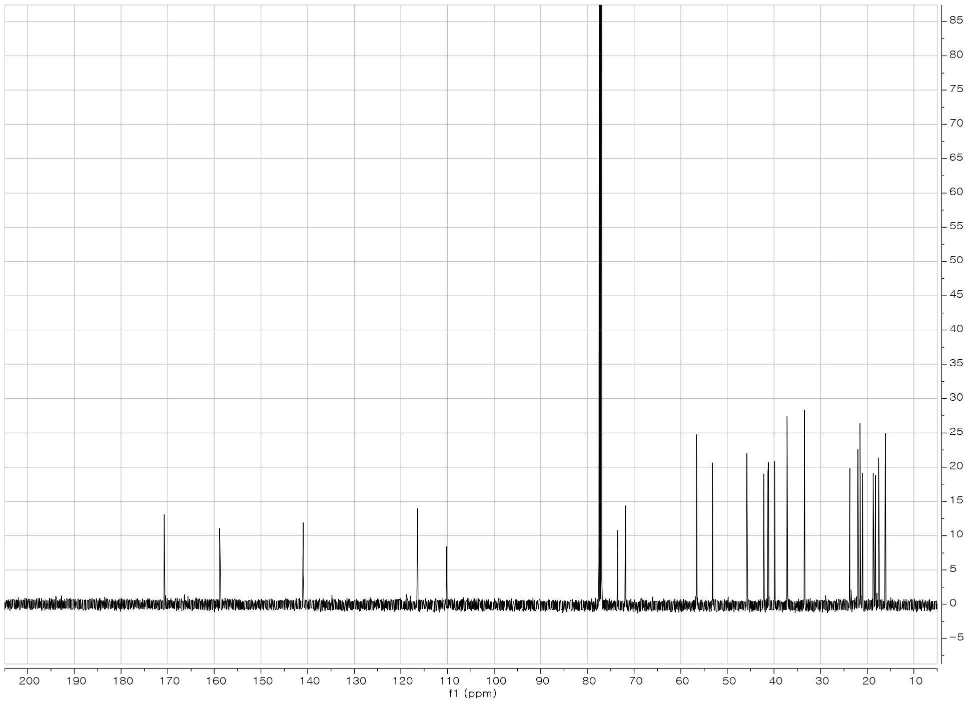

Figure S8. HMBC and HSQC NMR spectra of **5**

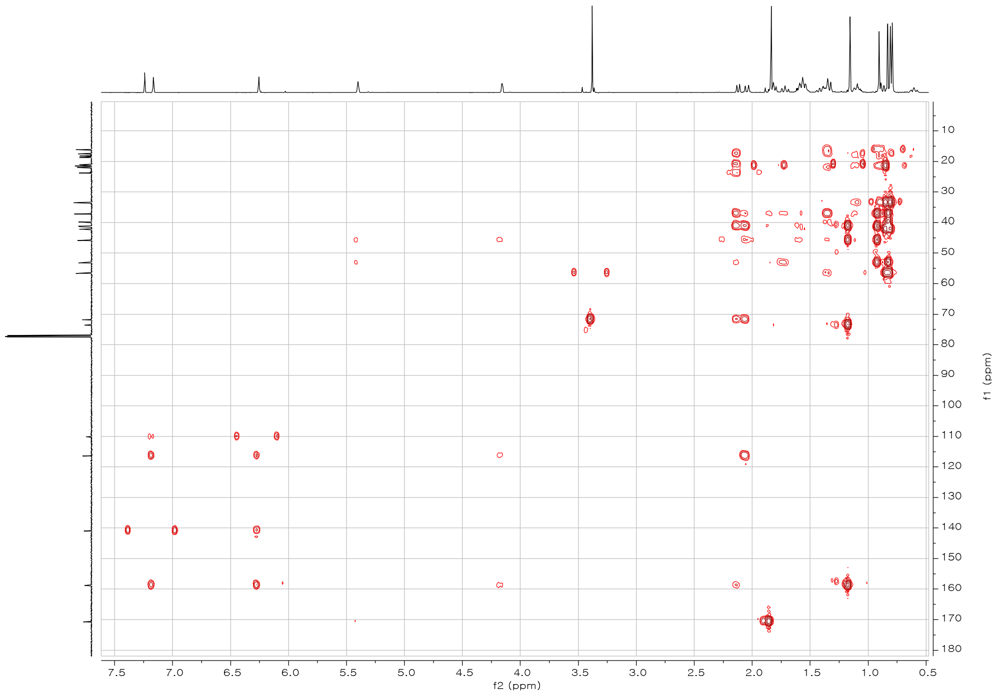

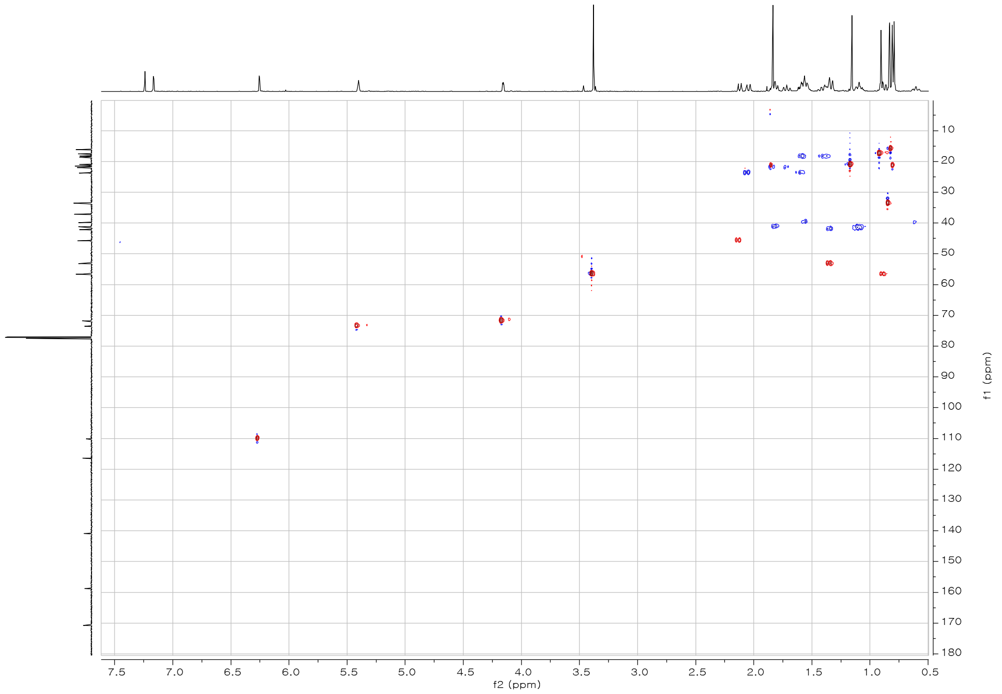

Figure S9. COSY and NOESY NMR spectra of **5**

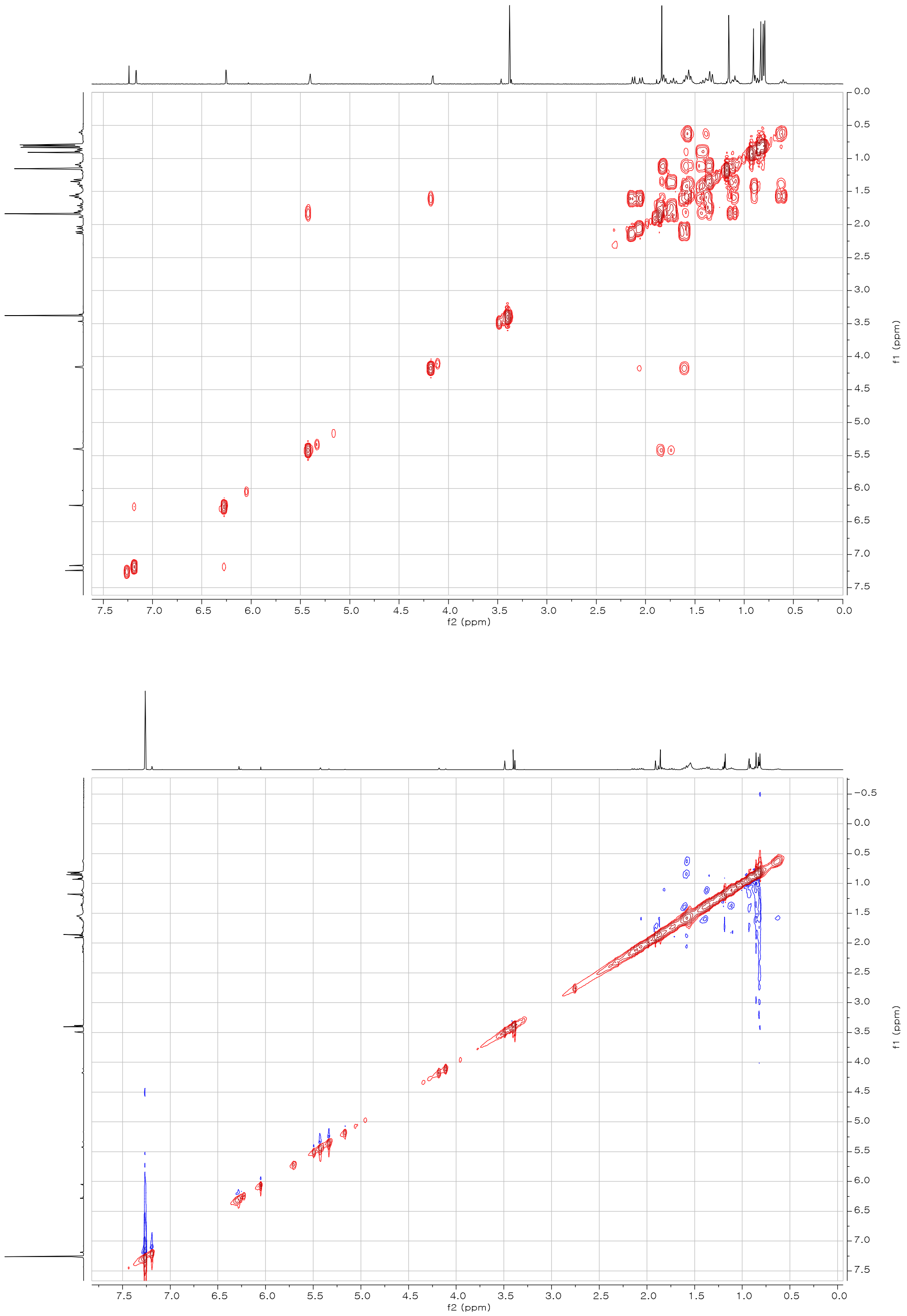

Figure S10. ^{1}H and ^{13}C NMR Spectra (500 MHz and 125 MHz, $CDCl_3$) of **1**

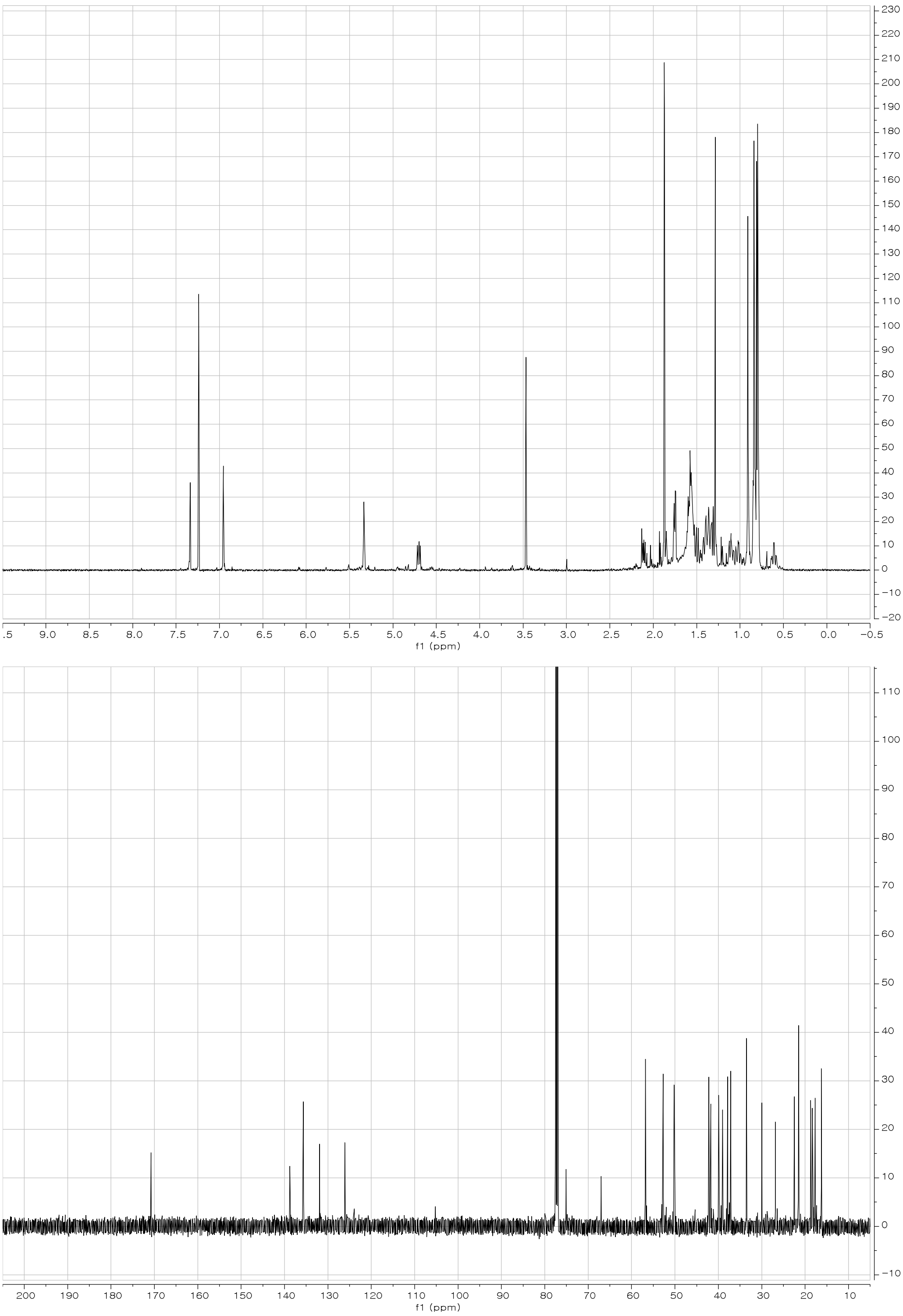

Figure S11. ^{1}H and ^{13}C NMR Spectra (500 MHz and 125 MHz, $CDCl_3$) of **2**

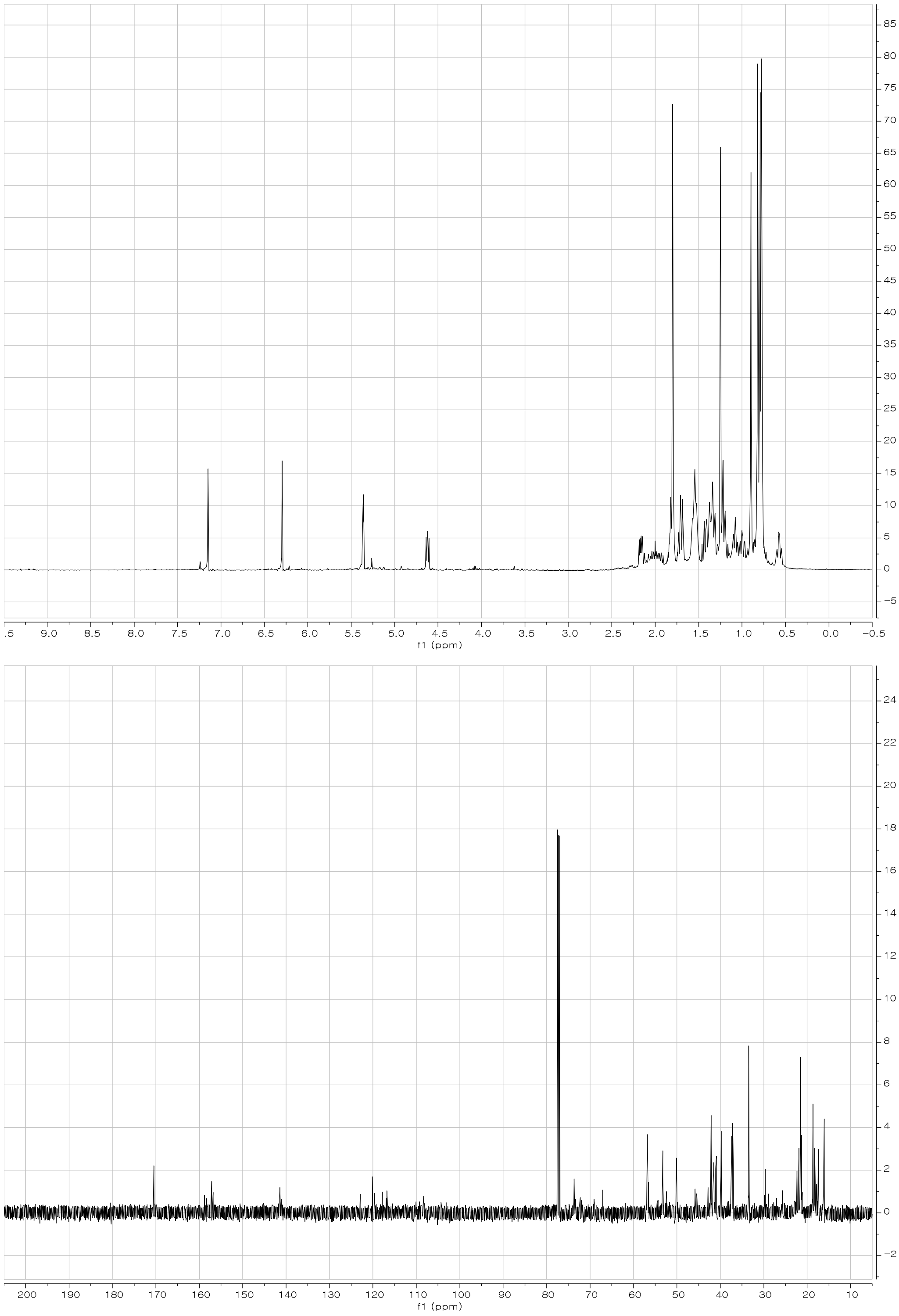

Figure S12. ^{1}H and ^{13}C NMR Spectra (500 MHz and 125 MHz, $CDCl_3$) of **6**

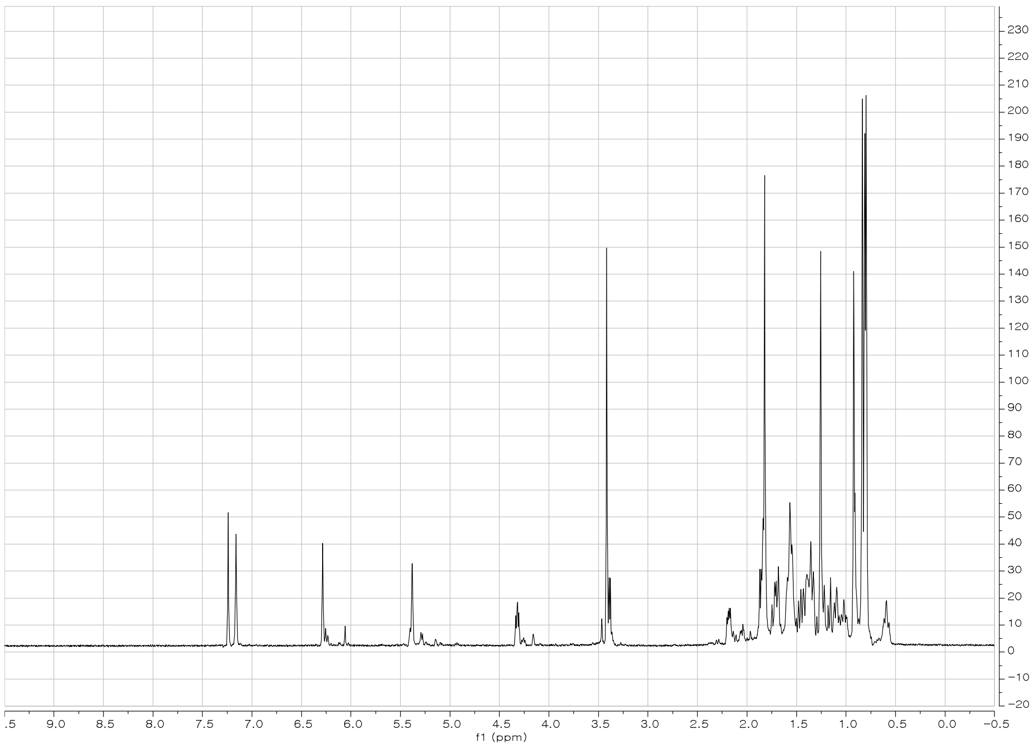
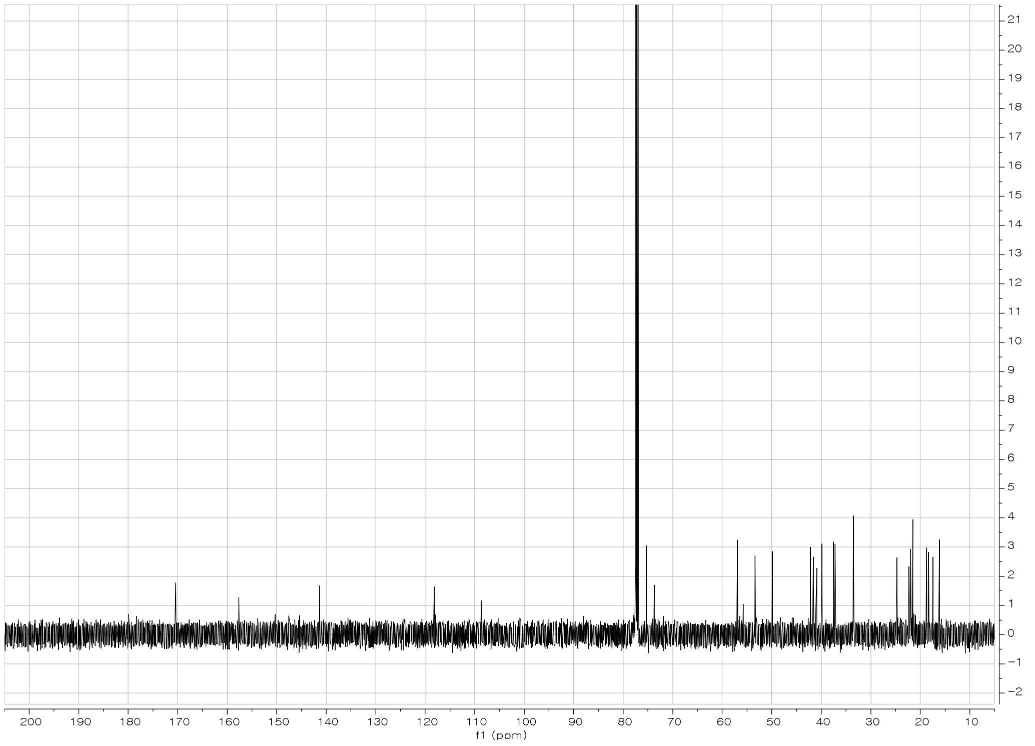

Figure S13. The comparison of ^{1}H and NMR Spectra (500 MHz, $CDCl_3$)

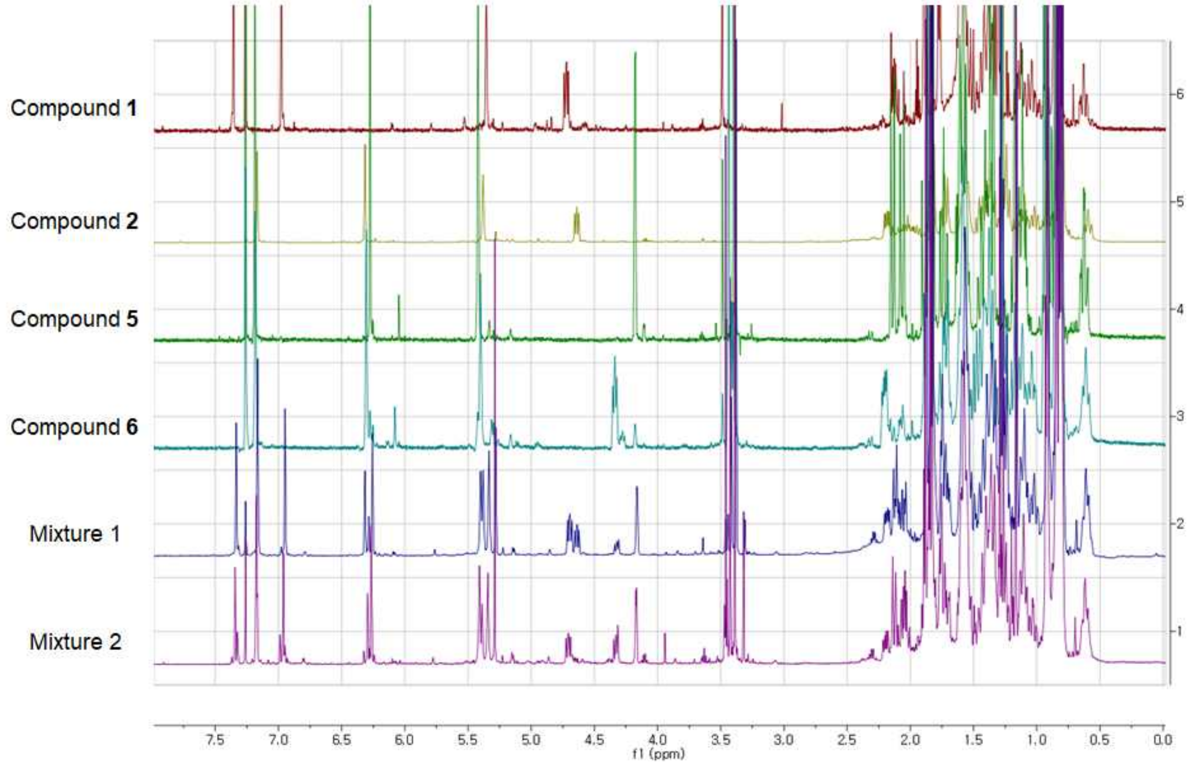

Mixture 1 : The fraction obtained by silica HPLC (methanol/dichloromethane) of the mixture of **1** and **2**

Mixture 2: The crude material obtained by the treatment of the mixture of **1** and **2** with silica and methanol for 60 min
